# Massachusetts Veterinary Medical Association

**Published:** 1885-01

**Authors:** W. Bryden, J F. Winchester

**Affiliations:** President; Secretary


### Proceedings Veterinary Medical Societies.

### MASSACHUSETTS VETERINARY MEDICAL ASSOCIATION.

At a meeting held at Young's Hotel, Boston, March 18, 1884, of veterinary surgeons practising in Massachusetts, it was voted to organize a State Society. There were fifteen gentlemen present, and F. H. Osgood, M.R.C.V.S., of Springfield, was chosen Chairman, and M. Bunker, D.V.S., of Newton, Secretary.

It was voted that the Charter Members shall be composed of graduates of regular veterinary schools, and that such gentlemen shall exhibit their diplomas to a committee hereafter to be appointed, for acceptance.

The following were chosen the committee: J. S. Saunders, D.V.S.; L. H. Howard, D.V.S.; W. Bryden, V.S.; F. S. Billings, V.S.; C. P. Lyman, F.R. C.V.S., of Boston. This committee to call the next meeting. Adjourned.

In conformity to the preliminary meeting, the committee called a meeting at the United States Hotel, April 2d, 1884, with F. H. Osgood, chairman, and M. Bunker, secretary, when they reported favorably in regard to all diplomas presented to them.

It was moved and carried that those of the original members who did not have their diplomas at the meeting of the committee be now examined.

It was also moved and carried that after this meeting no diplomas be examined by this committee.

The committee reported favorably on all diplomas except C. H. Hall's, which was not presented. The committee was then discharged.

It was moved and carried that we resolve ourselves into the Massachusetts Veterinary Association and ballot for President, Vice-President, Secretary, Treasurer and Executive Committe.

The ballot resulted in the election of the following officers: W. Bryden, V.S., of Boston, President; F. H. Osgood, M.R.C.V.S., Edinburgh, of Springfield, Vice-President; J. F. Winchester, D.V.S., of Lawrence, Secretary and Treasurer; F. S. Billings, V.S., of Boston, J. S. Saunders, D.V.S., of Boston, C. P. Lyman, F.R.C.V.S., of Boston, Executive Committee.

It was moved and carried that the Executive Committee draw up a set of by-laws and constitution, to be presented at the next meeting.

It was moved and carried that there be an assessment of one dollar on each member. Dr. Billings was appointed essayist for the next meeting.

THE SECOND MEETING of the Massachusetts Veterinary Association was held at the United States Hotel, May 7th, 1884, and was called to order at 7.45, with W. Bryden in the chair. Eleven members were present. Dr. Billings read a paper and a vote of thanks was tendered him.

Moved and carried that the Executive Committee present the Constitution and By-laws they had prepared, which were adopted as a whole.

Charles Byrne, M.R.C.V.S., W. T. Simmons, M.R.C.V.S., M.D., and Benjamin D. Pierce, M.R.C.V.S., presented their credentials.

F. S. Billings was appointed the next essayist; subject, "Homeopathy: a Contribution to the Code Question."

THE THIRD REGULAR MEETING of the Massachusetts Veterinary Association was held at the rooms of the Medical Library Association, 19 Boyleston Place, on Wednesday, June 4th, 1884, and was called to order at 8 P.M., with

W. Bryden in the Chair. There were twenty-three members present. Minutes of the last meeting read and approved.

Dr. Billings' paper was then read, for which, after a few remarks by Dr. Lyman, a vote of thanks was tendered him.

The Executive Committee reported having found the credentials of the following gentlemen satisfactory: T. J. O'Connell, V.S., Lynn; J. M. Skally, V.S., Boston; Chas. Byrne, M.R.C.V.S., North Cambridge; W. S. Simmons, M R.C.V.S., Boston; Benjamin D. Pierce, M.R.C.V.S., Springfield. They were balloted for and admitted.

F. S. Thomas, M.D., V.S., Hanson; A. W. Clement, V.S., Lawrence; J. S. Gardner, D.V.S., Greenfield; J. A. Soule, D.V.S., Hyde Park; J. B. Coleman, M.R.C.V.S., Boston; and J. H. Stickney, M.R.C.V.S., Boston, presented their names for admission.

Dr. Lyman was appointed the next essayist. Subject: "Art. I., Section 1."

THE FOURTH REGULAR MEETING of the Massachusetts Veterinary Association was held in Boston, 19 Boylestcn Place, Sept. 3d, and was called to order at 7.45 P. M., with W. Bryden in the chair.

Thirteen members answered the roll call, and the minutes of the last meeting were read and adopted.

Dr. Lyman moved, and it was seconded, that the name of the college from which each member of the Massachusetts Veterinary Association graduated be appended to his name. Carried.

The Executive Committee reported favorably on the credentials of F. S. Thomas of Hanson, W. A. Sherman of Lowell, J. S. Gardner of Greenfield, and Frederick Saunders of Lynn. They were then balloted for, and admitted as members of the Association.

Moved by Dr. Skally that the Executive Committee report upon a by-law to the effect that the reading of the essay follow immediately the reading of the minutes of the last meeting, and also to draw up a regular order of business. Seconded and carried.

Moved by Dr. Lyman that the Society add a *pro tem.* member to the Executive Committee during the absence of Dr. Billings. Seconded and carried.

Dr. Blackwood was nominated and elected a *pro tem.* member of the Executive Committee.

Dr. Lyman made a few remarks upon a case of contagious pleuro-pneumonia he was called to see in Brooklyn, and it was then moved and seconded that he read the report he had prepared instead of the paper on "Article I., Section 1." Carried. After a general discussion of the subject, Dr. Lyman was tendered a vote of thanks for his paper.

Dr. Billings sent in a paper to be opened and read at the meeting, and Dr. Lyman moved that it be referred to the Executive Committee. Lost.

Dr. Lyman moved that the further reading of Dr. Billings's resignation be suspended, for there had been no charges against him as a graduate of a Veterinary School by the Association. Amended by Dr. Osgood that the paper be submitted to those members of the Association appointed by the chair. Amendment carried.

Dr. Lyman moved that the further reading of that paper be postponed so far as the Association was concerned, and proceed to vote on the resignation of Dr. Billings. Lost.

Drs. Bunker, Osgood, and Howard were appointed a committee to report on Dr. Billings's paper.

Dr. Bunker reported that the paper was not necessary for the Association, but his resignation from the Executive Committee made known.

Dr. Billings's resignation from the Executive Committee was then accepted.

Dr. Blackwood was then elected a member of the Executive Committee.

Dr. Lyman, after a few remarks, tendered his resignation to the Massachusetts Veterinary Association.

Dr. Blackwood moved that Dr. Lyman's resignation be not accepted. Seconded and carried.

Dr. Coleman presented his credentials to the Executive Committee, and they were satisfactory.

J. M. Skally was appointed the next essayist. Subject: "Gangrene." Adjourned.

The Regular Meeting of the Massachusetts Veterinary Association, held Oct. 1, 1884, was called to order at 8 P. M., with W. Bryden in the chair.

Nine members answered the roll call, and the minutes of last meeting were read and adopted.

Dr. Lyman's resignation was accepted. It was moved and carried that the chair appoint a committee of three to bring in a name to fill the vacancy of the Executive Committee.

Drs. Simmons, Winchester, and F. Saunders were appointed, and Dr. Byrne's name presented.

Moved and carried that Dr. Byrne fill the vacancy on the Executive Committee.

Dr. Skally read a paper on "Gangrene," and after a general discussion of the subject a vote of thanks was tendered him.

Dr. Blackwood was appointed the next essayist. Meeting adjourned.

The Regular November Meeting of the Massachusetts Veterinary Association was held on the 5th, and called to order at 8 P. M., when the roll was called, twelve answering to it, and then the minutes of last meeting were read and adopted.

The Executive Committee reported an order of business, which was adopted.

Dr. Soule of Hyde Park applied for membership, but the Executive Committee reported unfavorably, and the Secretary was notified to communicate the fact to him. Charles Byrne was appointed next essayist. Subject: "Indigestion."

W. Bryden, President.

J F. Winchester, V.S., Secretary.

---

